# Supplementary material for: Genomic Landscape of Susceptibility to Severe COVID-19 in the Slovenian Population
Source: Int J Mol Sci. 2024 Jul 12;25(14):7674. doi: 10.3390/ijms25147674 (PMC11277002; doi:10.3390/ijms25147674)
Supplement: Supplementary file 1 [file ijms-25-07674-s001.zip › Supplementary table 1.pdf]

Supplementary Table 1. PICO (Participants, Interventions, Control, Outcomes) criteria was used for the inclusion and exclusion of screened studies.

PICO	Inclusion criteria	Exclusion criteria
**Participants**	COVID-19 positive diagnosis or/and level of severity of the disease	Participants with other infectious diseases and COVID-19 negative patients.
**Interventions**	Genotyping, sequencing, GWAS (genome wide association study), WES (whole exome sequencing), inclusive of any suitable method for the detectionof genetic variation, gene polymorphism, gene variant, allele(s), variant(s), host genetic(s), genetic susceptibility.	No genotype data
**Control**	COVID-19 negative or positive diagnosis or general population	No genotype data
**Outcomes**	SARS-CoV-2 infection, SARS-CoV-2 related comorbidities, requiring treatment in an intensive care unit, death	Other outcomes not related with SARS-CoV-2 infection

PICO: Participants, Interventions, Control, Outcomes; COVID-19: Coronavirus disease 19; GWAS: genome-wide association study; WES: whole exome sequencing; SARS-CoV-2: severe acute respiratory syndrome-2.
